# Differences in adults’ spatial scaling based on visual or haptic information

**DOI:** 10.1007/s10339-021-01071-0

**Published:** 2021-12-28

**Authors:** Magdalena Szubielska, Marta Szewczyk, Wenke Möhring

**Affiliations:** 1 Institute of Psychology, The John Paul II Catholic University of Lublin, Lublin, Poland; 2 Department of Educational and Health Psychology, University of Education Schwäbisch Gmünd, Schwäbisch Gmünd, Germany; 3 Faculty of Psychology, University of Basel, Basel, Switzerland

**Keywords:** Spatial cognition, Spatial scaling, Visual domain, Haptic domain

## Abstract

The present study examined differences in adults’ spatial-scaling abilities across three perceptual conditions: (1) visual, (2) haptic, and (3) visual and haptic. Participants were instructed to encode the position of a convex target presented in a simple map without a time limit. Immediately after encoding the map, participants were presented with a referent space and asked to place a disc at the same location from memory. All spaces were designed as tactile graphics. Positions of targets varied along the horizontal dimension. The referent space was constant in size while sizes of maps were systematically varied, resulting in three scaling factor conditions: 1:4, 1:2, 1:1. Sixty adults participated in the study (*M* = 21.18; *SD* = 1.05). One-third of them was blindfolded throughout the entire experiment (haptic condition). The second group of participants was allowed to see the graphics (visual condition); the third group were instructed to see and touch the graphics (bimodal condition). An analysis of participants’ absolute errors showed that participants produced larger errors in the haptic condition as opposed to the visual and bimodal conditions. There was also a significant interaction effect between scaling factor and perceptual condition. In the visual and bimodal conditions, results showed a linear increase in errors with higher scaling factors (which may suggest that adults adopted mental transformation strategies during the spatial scaling process), whereas, in the haptic condition, this relation was quadratic. Findings imply that adults’ spatial-scaling performance decreases when visual information is not available.

## Introduction

Spatial scaling of maps requires a comparison of different-sized spaces and demands an understanding of the connection between these spaces (Frick and Newcombe 2012). In order to investigate this ability, experimenters usually instruct participants to encode a simple map including a target and ask them to locate a target in an empty space at the same location (i.e., the referent space; Frick and Newcombe 2012; Hund et al. 2020; Huttenlocher et al. 1999; Möhring et al. 2014, 2015, 2018; Plumert et al. 2019; Vasilyeva and Huttenlocher 2004; but see Gilligan et al. 2018 for a discrimination task). Crucially, maps and the referent space differ in size so that participants need to scale spatial information from one space to the other.

Most spatial scaling studies have been conducted in the visual domain (Frick and Newcombe 2012; Gilligan et al. 2018; Hund et al. 2020; Huttenlocher et al. 1999; Möhring et al. 2014, 2015, 2016, 2018; Plumert et al. 2019; Vasilyeva and Huttenlocher 2004), with several studies indicating the usage of mental transformation strategies in adults (and children). Such a strategy was derived from increased errors and response times with higher scaling factors (e.g., Möhring et al. 2014, 2016); even though other results also exist (e.g., Frick and Newcombe 2012). Just recently, spatial scaling of maps was also tested in the haptic domain using convex graphics as stimuli (Szubielska et al. 2019; Szubielska and Möhring 2019). In these adult studies, it was found that absolute errors increased linearly with higher scaling factors, indicating similar mental transformation strategies. However, to our best knowledge, research has not yet compared spatial scaling across the visual and haptic domains using comparable methodologies and stimuli. Given that spatial information and maps can be processed in both domains (e.g., Craddock and Lawson 2009a, 2009b; Intraub et al. 2015; Klatzky et al. 1987; Loomis and Klatzky 2008; Szubielska and Balaj 2018), it seems crucial and timely to fill this gap and to increase our knowledge about spatial scaling of visually and haptically encoded maps.

Following the functional equivalence theory (Giudice et al. 2011), it seems likely that participants use similar size-scaling strategies in haptic and visual conditions and may not differ across conditions. However, it is also possible that participants show higher scaling performance in the visual as opposed to the haptic condition considering that haptic perception is slower and puts more strain on working memory than visual perception due to its sequential nature (Lederman and Klatzky 1987, 2009). As a consequence, maps may be encoded less accurately in the haptic than visual conditions (especially with a limited encoding time). Another possibility is that touch may have advantages over sight due to the possibility of using natural and accurate distance measures such as the length or width of a particular finger for encoding distances (Blanco and Travieso 2003), even though this finger-based strategy may interfere with using effective spatial-scaling strategies.

Moreover, spatial scaling of maps in a bimodal condition combining visual *and* haptic encoding may trump the spatial scaling process in the unimodal conditions, given that information can be encoded across two different senses. During this visuo-haptic perception, both modalities provide essential spatial information and may complement each other when being combined (Newell et al. 2001). According to previous literature (Ernst and Banks 2002; Ernst and Bülthoff 2004), the human brain efficiently merges information from vision and touch and processes them into a coherent percept. Whether vision or touch dominates in this integration process depends on the variance associated with the visual or haptic estimation. Perceptual processes seem to work in line with a general principle that minimalizes the variance in the final estimate. Summing up, the bimodal condition might lead to an even more accurate and robust percept of the target position than in the unimodal conditions.

The present study aimed to compare adults’ scaling ability of visually and haptically perceived maps, in addition to a bimodal condition combining visual *and* haptic perception. Based on the literature referenced above, we predicted that spatial scaling accuracy would be lower in the haptic than both in the visual condition and the bimodal condition (Hypothesis 1). We also hypothesized that for all three perceptual conditions, participants would produce larger errors with higher scaling factors, i.e., when the differences in size between a map and the reference space are larger (Hypothesis 2).

## Method

### Participants

A total of 60 undergraduate students (41 females, 19 males; 54 right-handed, 6 left-handed) aged between 19 and 23 years (*M* = 21.18, *SD* = 1.05) participated in the present study. Participants were randomly assigned to one of three perceptual conditions: (1) visual, (2) haptic, and (3) visual and haptic (*n* = 20 in each condition). There were no differences between the three experimental groups in terms of age, *F*(2,57) = 2.92, *p* = 0.062, gender, χ^2^(2) = 0.61, *p* = 0.735, or handedness, χ^2^(2) = 3.33, *p* = 0.189.

### Materials and procedure

**Fig. 1:**
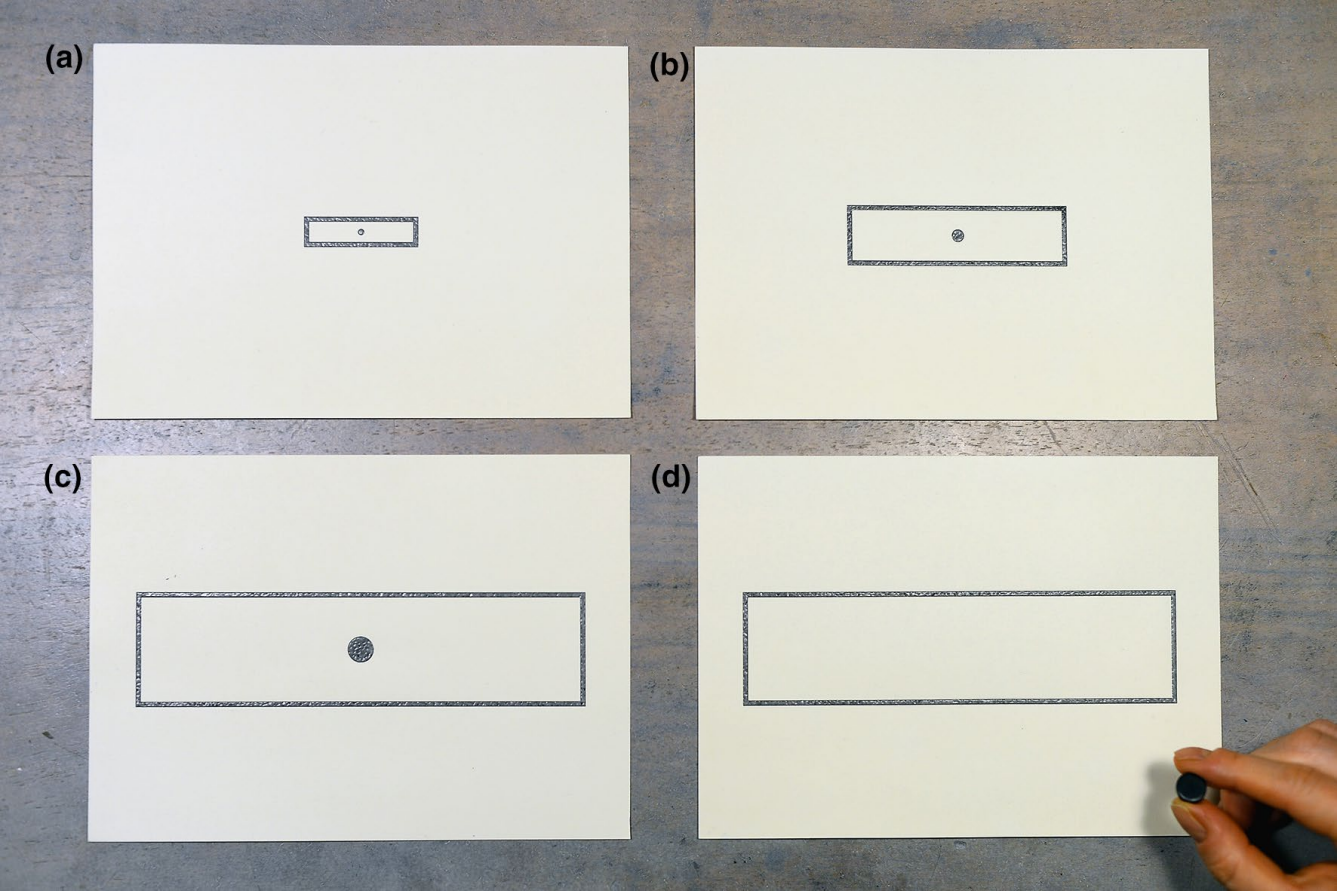
Example of maps for the scaling factors 1:4 (**a**), 1:2 (**b**), 1:1 (**c**), and a picture of giving a response in the reference space (**d**). The silver-gray elements of the boards are embossed

Materials were designed as boards (148.5 mm high × 210.0 mm wide) with tactile graphics, similar to materials used in the previous research in the field (Szubielska et al. 2019; Szubielska and Möhring 2019; for examples, see Fig. 1). There was one rectangular space on each board – representing either a map with a round target or an empty referent space (40.0 mm high × 170.0 mm wide). Maps were centered on the boards and varied in size (10.0 mm high × 42.5 mm wide; 20.0 mm high × 85.0 mm wide; 40.0 mm high × 170.0 mm wide) which corresponded to three scaling factor conditions (1:4, 1:2, and 1:1). Target locations varied on the horizontal dimension (see Table 1) and targets differed in size (diameters: 2.5 mm, 5.0 mm, 10.0 mm) in accordance with the scaling factor conditions. Target locations (7) and scaling factors (3) were combined using a full-factorial design, amounting to a total of 21 trials for each participant. Thus, target location and scaling factor served as within-subject variables whereas perceptual condition served as a between-subjects variable.

**Table 1:** Correct target locations on the referent space (in mm)

Target location	Y-coordinate	X-coordinate
1 (L1: first from the left)	20	17.5
2 (L2: second from the left)	20	40
3 (L3: third from the left)	20	62.5
4 (M: in the middle of the field)	20	85
5 (R3: third from the right)	20	107.5
6 (R2: second from the right)	20	130
7 (R1: first from the right)	20	152.5

Participants were tested individually in a single session. Boards including the map were presented in a random order one after the other by placing them at the top of the table where the participant sat. Each test trial followed the same structure: First, participants were instructed to encode the position of a target on the map without a time limit using (1) sight, (2) manual exploration, (3) or both (depending on conditions). The way of manually exploring the map was not determined. Hence, some participants used the dominant hand while others used two hands or just selected fingers for the exploration. In the bimodal condition, participants were instructed to use both vision and touch for learning the stimuli (although participants followed this instruction, the experimenter observed that in the bimodal condition, they explored the maps less thoroughly by touch than in the haptic condition). Afterward, the map was removed from the table, and participants were presented with the constant-sized referent space. They were then asked to locate a target in this referent space using a round disc (with a diameter of 10 mm) from memory.

In the haptic perceptual condition, participants were blindfolded during the test phase and were allowed to touch the map when the experimenter said "now". In the visual and bimodal conditions, participants were asked to close their eyes and open them only when the experimenter said "now"—when the particular map was placed in front of the participant. Participants signaled their learning of the target’s location by saying “ready”. The time of encoding the target location was measured by the experimenter using a stopwatch from the "now" signal of the experimenter until the participant said "ready". After participants placed the round disc at the same location in the referent space, the experimenter measured accuracy for each response (in mm) by noting the x- and y-coordinates.

## Results

*Data preparation.* Absolute errors were computed based on the x- and y-coordinates, using the Euclidean distance formula (the distance in a straight line between the participant’s response and the correct target location). In addition, signed errors were calculated because they inform us about the precision of responses, namely—the spread of the responses on the horizontal dimension (cf. Frick and Newcombe 2012). We computed them by subtracting the x-coordinate of the respective target locations from the x-coordinate of the participant’s responses. Thus, positive values of signed errors indicate that responses were located too far to the right on the referent space; negative values of signed errors demonstrated that responses were located too far to the left. Outliers (*M* ± 3 *SD*s) in participants’ absolute errors, signed errors, and encoding times were identified and excluded. We identified outliers in 1.90% of all cases (from a total of 1260 responses) with respect to absolute errors, 1.65% cases of signed errors, and 1.27% cases of the encoding times. Data were collapsed across all trials of each participant, but separately for each scaling factor (1:4, 1:2, 1:1) and dependent variable (absolute errors, signed errors, encoding times).

**Fig. 2:**
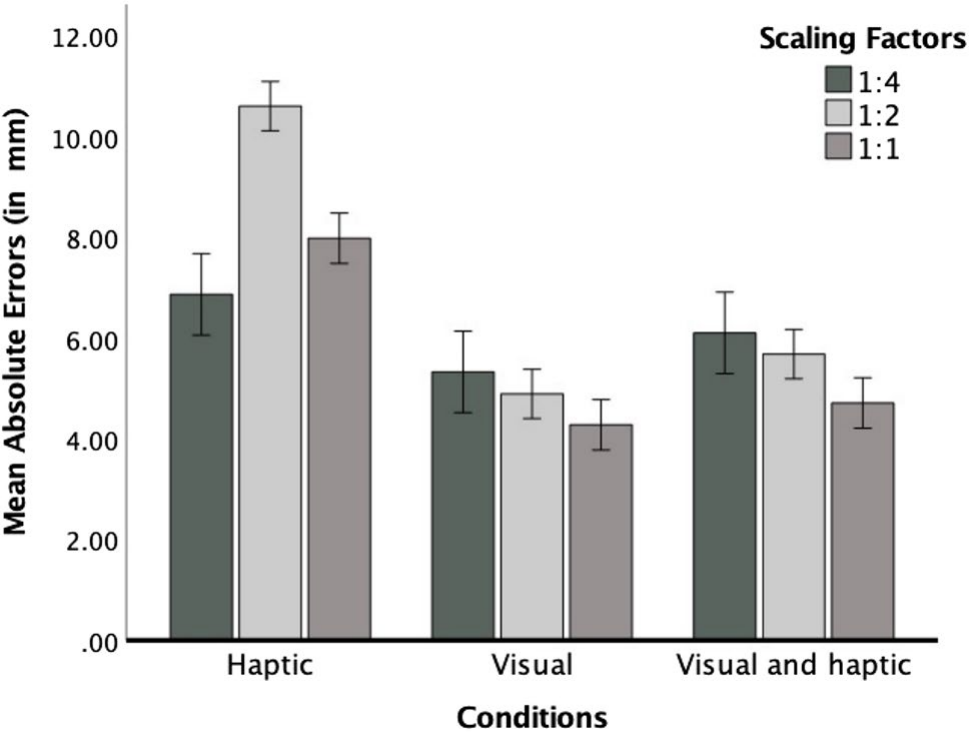
Mean value of absolute errors as a function of scaling factor in three perceptual conditions. Error bars represent ± 1 standard error

*Absolute errors.* We calculated repeated measures analysis of variance (ANOVA) with scaling factor (3) as a within-subject variable, perceptual condition (3) as a between-subjects variable, and absolute errors as a dependent variable. Greenhouse–Geisser corrections were used whenever necessary to account for violations of the sphericity assumption, and significant effects in ANOVAs were followed up by post hoc comparisons using Bonferroni adjustments (in this and the following ANOVAs). The analysis yielded a significant main effect of perceptual condition, *F*(2, 57) = 17.90, *p* < 0.001, η_p_^2^ = 0.39, because participants produced larger errors in the haptic condition (*M* = 8.25, *SE* = 0.43) than in the visual (*M* = 4.84, *SE* = 0.43) and bimodal condition (*M* = 5.50, *SE* = 0.43) (both *p*s < 0.001). The difference between the visual and the bimodal condition was not significant (*p* = 0.825). The ANOVA also revealed a significant effect of scaling factor, *F*(1.63, 93.64) = 8.91, *p* = 0.001, η_p_^2^ = 0.14, that was qualified by a significant interaction between scaling factor and perceptual condition, *F*(3.29, 93.64) = 10.57, *p* < 0.001, η_p_^2^ = 0.27. We computed similar, separate repeated measures ANOVAs as indicated above for each perceptual condition to investigate this interaction. For the haptic condition, the effect of scaling factor reached significance, *F*(1.49, 28.28) = 14.84, *p* < 0.001, η_p_^2^ = 0.44, and was best described by a significant quadratic function, *F*(1, 19) = 25.42, *p* < 0.001, η_p_^2^ = 0.57. In this condition, errors increased between the scaling factors conditions 1:1 and 1:2, and then, decreased between the scaling factor conditions 1:2 and 1:4 (see Fig. 2). For the bimodal condition, the effect of scaling factor was significant, *F*(2, 38) = 3.49, *p* = 0.040, η_p_^2^ = 0.16, and was best described by a significant linear function, *F*(1, 19) = 4.49, *p* = 0.047, η_p_^2^ = 0.19, because absolute errors increased with higher scaling factors (see Fig. 2). For the visual condition, the effect of scaling factor tended towards significance but revealed a relatively high effect size, *F*(2, 38) = 2.87, *p* = 0.069, η_p_^2^ = 0.13. This effect was best described by a linear function which again tended towards significance, *F*(1, 19) = 4.08, *p* = 0.058, η_p_^2^ = 0.18. It was found that absolute errors increased with higher scaling factors (see Fig. 2). After combining data from both visual conditions (i.e., visual and bimodal), the ANOVA yielded a significant effect of scaling factor, *F*(1.62, 63.32) = 6.46, *p* = 0.005, η_p_^2^ = 0.14, that was best described by a significant linear function, *F*(1, 39) = 8.68, *p* = 0.005, η_p_^2^ = 0.18, because absolute errors were larger for the higher scaling factors (*M*_1:4_ = 5.72, *SE*_1:4_ = 0.37 vs. *M*_1:2_ = 5.29, *SE*_1:2_ = 0.25 vs. *M*_1:1_ = 4.50, *SE*_1:1_ = 0.32).

*Signed errors.* We calculated repeated measures ANOVA with scaling factor (3) and target location (7) as within-subject variables, perceptual condition (3) as a between-subjects variable, and signed errors as a dependent variable. This analysis revealed that all main (except perceptual condition), as well as interaction effects were significant (see Table 2 for details). Given these results, and in particular the significant three-way interaction effect, we decided to conduct nine repeated measures ANOVAs with target location (7) as a within-subject variable, separately for each level of scaling factor (3) and perceptual condition (3). The main effect of target location was significant in all nine analyses (all *F*s > 4.10, all *p*s > 0.01). Therefore, it seems that in each perceptual and scaling factor condition, participants showed some bias when locating the targets.

**Table 2:** Results of ANOVA on signed errors: all main and interaction effects

Effect	df_Num_	df_Den_	*F*	*p*	η_p_^2^
Scaling factor	2	96	4.14	.019	.08
Perceptual condition	2	48	2.16	.126	.08
Target location	3.82	183.53	15.63	< .001	.25
Scaling factor x Perceptual condition	4	96	2.83	.029	.10
Scaling factor x Target location	7.82	375.64	15.08	< .001	.24
Target location x Perceptual condition	12	288	30.35	< .001	.56
Scaling factor x Perceptual condition x Target location	24	576	6.30	< .001	.21

Considering that we were most interested in comparing performance across the three perceptual conditions, we subsequently analyzed the results collapsed across all levels of scaling factors. We performed seven separate univariate ANOVAs (for each target location) with perceptual condition (3) as a between-subjects factor and the signed errors as the dependent variable. The main effect was significant in almost all analyses, except from the middle (M) as well as the R2 target locations (see Table 3 for details). Thus, it seems that participants of all three perceptual conditions were able to locate the mid-position (and another target location) with comparable accuracy.

**Table 3:** Results of seven separate univariate ANOVAs on signed errors: main effects of perceptual condition

Target location	df_Num_	df_Den_	*F*	*p*	η_p_^2^
L1	2	57	11.03	< .001	.28
L2	2	57	6.74	.002	.19
L3	2	57	33.27	< .001	.54
M	2	57	.34	.71	.01
R3	2	57	13.94	< .001	.33
R2	2	57	2.42	.10	.08
R1	2	57	75.45	< .001	.73

**Fig. 3:**
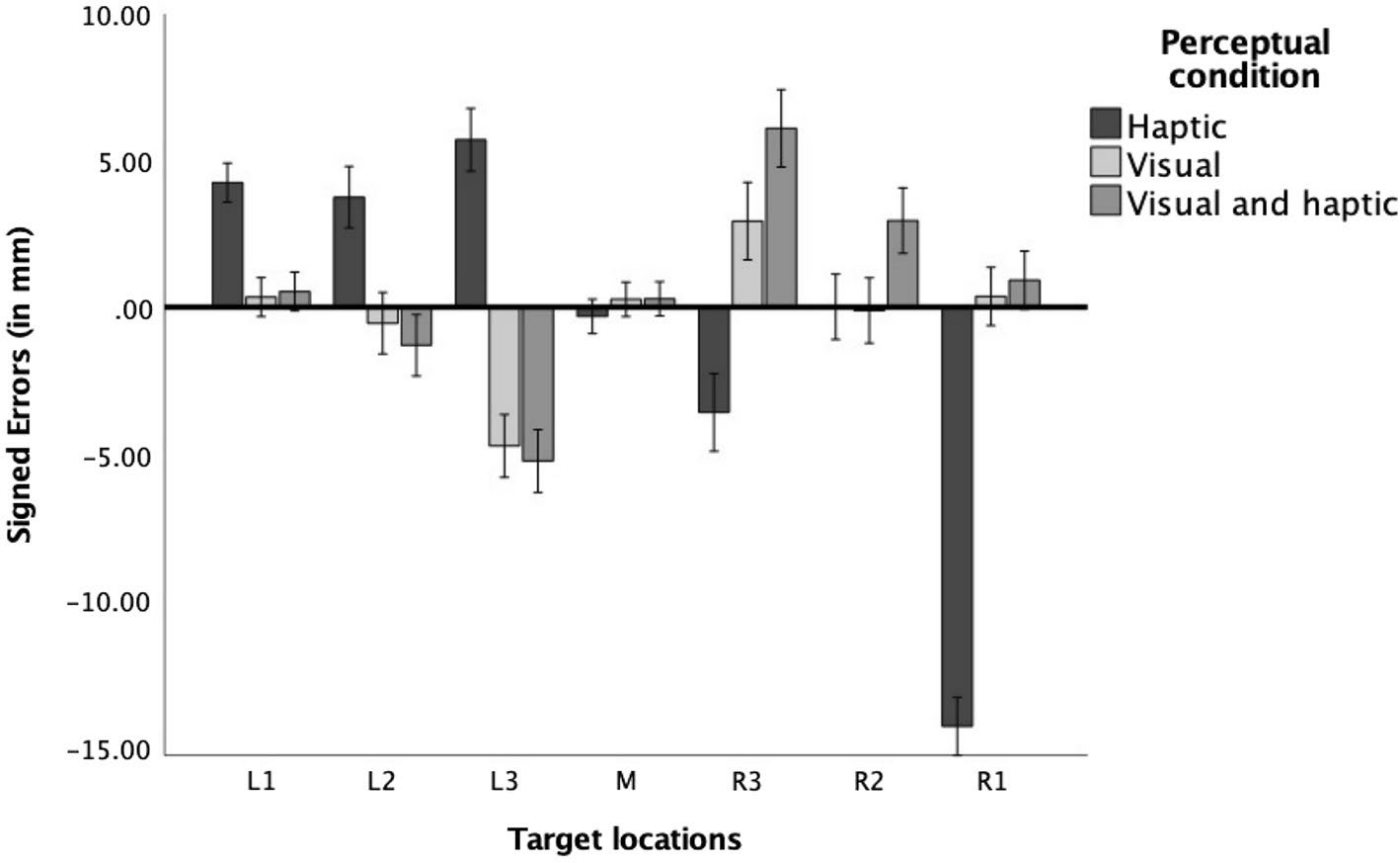
Signed errors for each target location in the three perceptual conditions, collapsed across the scaling factor conditions. Error bars represent ± 1 standard error*.* Positive values of signed errors indicate that responses were located too far to the right on the referent space; negative values of signed errors demonstrated that responses were located too far to the left

Finally, to explore the pattern of results for each perceptual condition across all target locations, we performed a repeated measures ANOVA with target position as a within-subject factor and perceptual condition as a between-subjects factor and signed errors as the dependent variable. The main effect of target location was significant, *F*(6,342) = 14.81, *p* < 0.001, η_p_^2^ = 0.21, and this effect was qualified by the interaction between target location and perceptual condition, *F*(12,342) = 26.10, *p* < 0.001, η_p_^2^ = 0.48. Contrast analyses revealed that errors in the haptic condition were best explained by the linear function, *F*(1,19) = 54.22, *p* < 0.01, η_p_^2^ = 0.29, whereas errors in the visual and bimodal conditions were better explained by the cubic function *F*(1,19) = 83.91, *p* < 0.001, η_p_^2^ = 0.74. As can be seen in Fig. 3, participants’ answers in the haptic condition seemed to gravitate towards the middle, whereas participants’ answers in the visual and bimodal condition seemed to gravitate towards an imagined midpoint on the left and right half of the referent space.

**Fig. 4:**
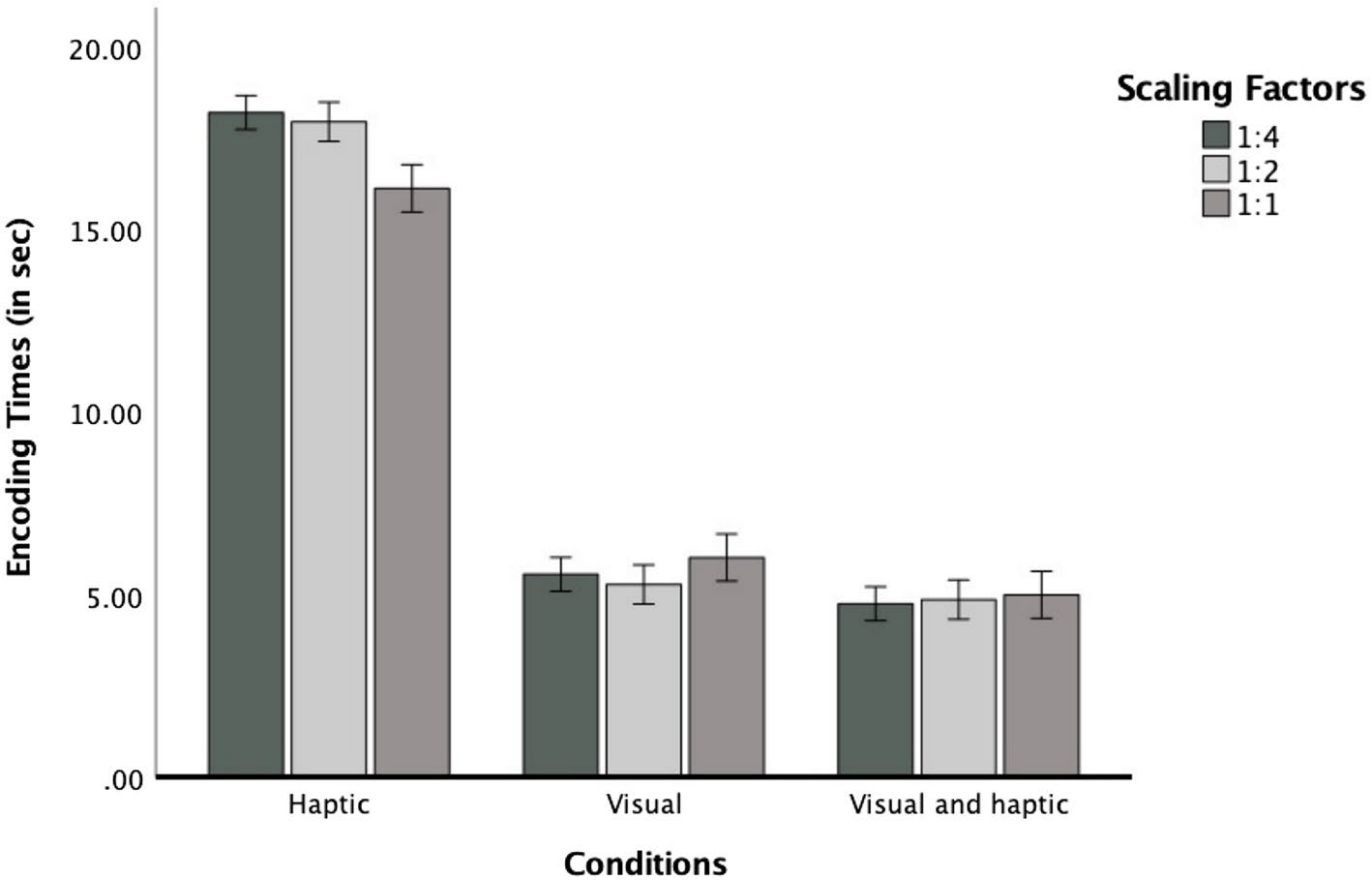
Mean value of encoding times as a function of scaling factor in three perceptual conditions. Error bars represent ± 1 standard error

*Encoding times.* We calculated a repeated measures ANOVA with scaling factor (3) as a within-subject variable, perceptual condition (3) as a between-subjects variable, and encoding times as the dependent variable. The main effect of perceptual condition reached significance, *F*(2, 57) = 234.37, *p* < 0.001, η_p_^2^ = 0.89, because participants encoded maps for a longer time in the haptic condition (*M* = 17.41, *SE* = 0.46) than in the visual (*M* = 5.61, *SE* = 0.46) and the bimodal condition (*M* = 4.87, *SE* = 0.46) (both *p*s < 0.001). The difference between both visual conditions was not significant (*p* = 0.764). The main effect of scaling factor was not significant, *F*(2, 114) = 1.15, *p* = 0.320, but the interaction between perceptual condition and scaling factor reached significance, *F*(4, 114) = 4.43, *p* = 0.002, η_p_^2^ = 0.13. To investigate this interaction, we calculated a repeated measures ANOVA for each perceptual condition. For the haptic condition, the main effect of scaling factor was significant, *F*(2, 38) = 4.54, *p* = 0.017, η_p_^2^ = 0.19, and best described by a significant linear function, *F*(1, 19) = 7.14, *p* = 0.015, η_p_^2^ = 0.27, showing that participants encoded maps longer with increasing scaling factor (see Fig. 4). The effect of scaling factor was not significant in the visual, *F*(2, 38) = 1.36, *p* = 0.269, nor the bimodal condition, *F*(2, 38) = 0.32, *p* = 0.727.

## Discussion

The current study aimed at testing differences between spatial scaling in the visual and tactile domains. Participants perceived maps with different sizes visually and/or haptically and gave responses from memory on the empty referent space with a fixed size. As predicted, in the haptic condition, participants produced larger errors in the spatial localization task than in both visual conditions, supporting Hypothesis 1. Hence, our results seem to contradict the functional equivalence theory (Giudice et al. 2011) since memorizing the map and updating performance (i.e., scaling up information from the recently encoded map) were more accurate in both visual conditions than in the haptic condition. At the same time, participants were not more accurate in the bimodal condition than in the visual condition. Furthermore, participants encoded maps longer in the haptic condition than in both visual conditions. This behavior might have been caused by lower processing speed in tactile perception than in visual perception (Lederman and Klatzky 1987, 2009) and time-consuming attempts to translate the haptic code into a visual code (because sighted individuals tend to represent spatial information in the visual form even if the source of information is not visual: Pantelides et al. 2016; Szubielska 2014; Szubielska and Zabielska-Mendyk 2018; Vanlierde and Wanet-Defalque 2004). Hence, even though participants encoded maps for a longer time in the haptic condition, these encoding times did not necessarily translate into more accurate spatial scaling from memory. It may have been difficult to build a holistic mental representation of the map based on haptic perception. Spatial cognition through touch is more demanding than visual cognition (Lederman and Klatzky 2009; Morimoto 2020; Yoshida et al. 2015), which might result in the creation of less accurate mental representations of maps. Moreover, we tested sighted individuals who do not need to measure with their fingers/hands on a daily basis (as opposed to blind people, who do this frequently: Blanco and Travieso 2003).

This may also explain why the bimodal condition was not beneficial for sighted participants. Despite being able to assess distances and measure with the hand, participants may have relied mainly on visual information in this bimodal condition. This can be concluded based on the similarity in participants’ response patterns in absolute and signed errors as well as encoding times between this bimodal condition and the visual condition, as opposed to the haptic condition. Based on this latter result and considering that participants produced more accurate responses in the visual condition than in the haptic condition, one may conclude that the visual information was more dominant in our task, and that information originating from visual and haptic modalities were not fully integrated by participants in the bimodal condition. However, the present data and the used methodology do not allow to draw firm conclusions about information integration. First, we used a between-participants design. Second, we did not measure reaction time, which is a reliable measure of accessing how spatial information is stored in memory and may indicate different aspects about information integration in working memory as opposed to accuracy measures (Pantelides et al., 2016). Third, we did not systematically vary information on the visual and haptic dimensions of the bimodal condition, which would be necessary in order to assess the dominance of each perceptual sense in adults’ responses (Wilkening et al. 1980).

We also predicted a linear increase in absolute errors with increasing scaling factors for all three perceptual conditions (Hypothesis 2). This hypothesis was supported in the visual and bimodal conditions even though in the visual condition, the result was only close to statistical significance. When data of both visual conditions (visual and bimodal) were combined, the result was significant and in line with the hypothesis. In the haptic condition, the relationship between the increase in scaling factor and errors was quadratic because errors were larger for scaling factors of 1:2 than 1:1 and 1:4. Considering that we assumed that accuracy in the location task depends on the accuracy of encoding maps and spatial scaling ability, this quadratic relationship might refer to the longer encoding times in the 1:4 scaling factor condition. It is possible that participants encoded the maps most accurately in this scaling factor condition given the need to explore these smallest maps more closely due to their perceived small size. Therefore, errors in the spatial localization task do not allow us to draw clear conclusions about the spatial scaling strategies used by participants in the haptic condition. On the contrary, participants’ errors in both visual conditions suggest the usage of mental transformation strategies even though it would be preferable to look at errors as well as response times as indicators of mental transformation strategies (Gilligan et al. 2018; Möhring et al. 2014, 2016; Szubielska and Möhring 2019).

Furthermore, we observed differences in the pattern of signed errors between the haptic and visual modalities. Namely, we saw a linear function between the horizontal target location and signed errors when the target was perceived haptically, indicating that participants tended to gravitate towards the middle of the perceptual space in this condition. At the same time, this function was cubic for the performance of participants in the visual and bimodal conditions. In line with findings from Plumert et al. (2019; see also Hund et al. 2020), this may indicate that participants split the space into two spaces (left and right) and gravitated towards the center of each half in the visual conditions (see also Huttenlocher et al. 1994). In accordance with the category adjustment model (Huttenlocher et al. 1991), encoding space haptically may refrain participants from using more fine-grained categories when exploring spatial layouts, and instead, they may use the entire space as one single entity. Such behavior may ultimately result in lower accuracy when localizing targets, which is supported by our data.

Our study provides a first step into comparing spatial scaling between the visual and haptic modalities; however, several limitations warrant mention. First, to draw conclusions about the usage of scaling strategies, it would be preferable to systematically manipulate scaling factors and measure errors and response times (Gilligan et al. 2018; Möhring et al. 2014). Unfortunately, in the current study, response times were not measured. Second, if the encoding times were not unlimited, results could differ. Recent research often used a fixed encoding time, with times being longer for the haptic condition than for the visual condition (e.g., Ernst et al. 2007; Newell et al. 2005; Szubielska and Bałaj 2018). Third, it was not determined in the present study how participants manually explored the maps. The participants were free to choose their preferred strategy of exploring the spatial layout—which may have increased the variance in the accuracy of the haptic condition. Fourth, we are unable to disentangle times of haptic exploring the spatial layout and the encoding times in the current study. Due to its sequential nature, exploration times should be longer in the haptic condition than in the visual ones and this affects the measure of encoding time, especially in the haptic condition. Fifth, the study involves fewer male participants as compared to females. Since spatial scaling ability seems to differ between males and females with a moderate effect size (Yuan et al. 2019), the findings of the current study should be generalized with caution.

To conclude, in the current study, we have compared the accuracy of spatial scaling in the visual and/or haptic conditions for the first time. Results showed that spatial-scaling performance decreased considerably when adult participants were blindfolded during the tests as compared to the conditions that allowed participants to explore the map and the referent space by sight. The present findings may provide a first step into increasing our understanding of how scaling differs between different perceptual modalities and may have high practical significance for developing effective spatial scaling training for blind individuals who can only rely on haptic encoding.

## Data Availability

The research data is available per request at the corresponding author's email address (magdasz@kul.pl).
